# *AHRR* (cg05575921) hypomethylation marks smoking behaviour, morbidity and mortality

**DOI:** 10.1136/thoraxjnl-2016-208789

**Published:** 2017-01-18

**Authors:** Stig E Bojesen, Nicholas Timpson, Caroline Relton, George Davey Smith, Børge G Nordestgaard

**Affiliations:** 1 Department of Clinical Biochemistry, Herlev and Gentofte Hospital, Copenhagen University Hospital, Herlev, Denmark; 2 Faculty of Health and Medical Sciences, University of Copenhagen, Copenhagen, Denmark; 3 The Copenhagen City Heart Study, Frederiksberg Hospital, Copenhagen University Hospital, Copenhagen, Denmark; 4 MRC Integrative Epidemiology Unit (IEU), School of Social and Community Medicine, University of Bristol, Bristol, UK

**Keywords:** Tobacco and the lung, Lung Cancer, COPD epidemiology

## Abstract

**Rationale and objectives:**

Self-reported smoking underestimates disease risk. Smoking affects DNA methylation, in particular the cg05575921 site in the aryl hydrocarbon receptor repressor *(AHRR*) gene. We tested the hypothesis that *AHRR* cg05575921 hypomethylation is associated with risk of smoking-related morbidity and mortality.

**Methods:**

From the Copenhagen City Heart Study representing the Danish general population, we studied 9234 individuals. Using bisulphite treated leucocyte DNA, *AHRR* (cg05575921) methylation was measured. Rs1051730 (*CHRN3A*) genotype was used to evaluate smoking heaviness. Participants were followed for up to 22 years for exacerbations of COPD, event of lung cancer and all-cause mortality. Six-year lung cancer risk was calculated according to the Prostate, Lung, Colorectal and Ovarian Cancer Screening Trial (PLCO_M2012_).

**Measurements and main results:**

*AHRR* (cg05575921) hypomethylation was associated with former and current smoking status, high daily and cumulative smoking, short time since smoking cessation (all p values <7×10^–31^), and the smoking-related *CHRN3A* genotype (−0.48% per T-allele, p=0.002). The multifactorially adjusted HRs for the lowest versus highest methylation quintiles were 4.58 (95% CI 2.83 to 7.42) for COPD exacerbations, 4.87 (2.31 to 10.3) for lung cancer and 1.67 (1.48 to 1.88) for all-cause mortality. Finally, among 2576 high-risk smokers eligible for lung cancer screening by CT, observed cumulative incidences of lung cancer after 6 years for individuals in the lowest and highest methylation quintiles were 3.7% and 0.0% (p=2×10^–7^), whereas predicted PLCO_M2012_ 6-year risks were similar (4.3% and 4.4%, p=0.77).

**Conclusion:**

*AHRR* (cg05575921) hypomethylation, a marker of smoking behaviour, provides potentially clinical relevant predictions of future smoking-related morbidity and mortality.

**Key messages**

**What is the key question?**

- Is the extent of *AHRR* cg05575921 hypomethylation in leucocyte DNA associated with risk of smoking-related morbidity and mortality? If so, does such hypomethylation provide information beyond that of self-reported (subjective) smoking information?

**What is the bottom line?**

- Using DNA blood samples drawn in 1991–1994 from individuals in the Copenhagen City Heart Study, representing the Danish general population, we followed 9234 individuals for COPD exacerbations, lung cancer and all-cause mortality, and found that AHRR cg05575921 hypomethylation strongly predicted risks, also after adjustment for self-reported smoking behaviour at the time of blood draw.

**Why read on?**

- Because tobacco smoking remains an important cause of preventable morbidity and mortality, but self-reported smoking behaviour underestimates disease risk, this novel objective marker of long-term smoking behaviour could be of clinical value if included in the risk calculation algorithms identifying high-risk smokers for screening or other interventions.

## Introduction

Tobacco smoking causes morbidity leading to disabilities and reduced life quality, and causes 12% of all deaths globally.^1^ Self-reported smoking information is subject to recall bias and understatement by the patient.^2^ ^3^ Furthermore, it does not capture subtle, but important variation in smoking behaviour due to variation of number of inhalations per cigarette, depth of inhalations, respiratory retention of the smoke and heaviness of second-hand on top of first-hand smoking.^4^ ^5^ Therefore, self-reported cross-sectional smoking information produces underestimated risk estimates, as suggested in genetic studies using a smoking-related genotype of nicotinic acetylcholine receptor (*CHRNA3*) as proxy for the lifetime exposure to tobacco smoke^6^; however, in that study, association of genotype with cigarette consumption was based on current smoking status and not on lifetime exposure and was not adjusted for factors that influence nicotine metabolism.

Objective measures of smoking behaviour consist mainly of measurements in body fluids of cotinine^7^ or exhaled CO concentration.^8^ With biological half-lives below 24 hours, these measurements are, however, not well-suited to estimate long-term smoking behaviour.

In CpG sites across the genome, methylation extent is associated with tobacco smoking.^9^ Of these, one situated in intron 3 of the aryl hydrocarbon receptor repressor (*AHRR*) gene on chromosome 5 (annotated on the Illumina HumanMethylation450 BeadChip array as probe cg05575921), showed greatest evidence for different methylation in response to exposure to tobacco smoking, as found by others,^10–15^ and in a recent meta-analysis.^16^ *AHRR* (cg05575921) methylation extent is also inversely correlated with serum cotinine,^11^ exhaled CO concentration and reverts after smoking cessation.^17^

Smoking-responsive DNA methylation changes at other loci are associated with risk of lung cancer and mortality^18^ ^19^; however, it is not known whether variation in *AHRR* (cg05575921) methylation extent is associated with risk of future smoking-related morbidity and mortality among individuals from the general population, and how useful this association might be for (pre-)clinical management of smokers. Screening with low-dose CT scans reduces lung cancer mortality among individuals with high a priori risk, but only marginally among those at lowest risk.^20^ Therefore, several algorithms, which include self-reported smoking have recently been developed to predict smokers' risk of lung cancer.^21^ ^22^ Measurement of *AHRR* methylation extent might, therefore, provide valuable information beyond that of existing risk prediction models identifying individuals eligible for CT scan lung cancer screening, for example the 6-year predicted risk algorithm (PLCO_M2012_) from the Prostate, Lung, Colorectal and Ovarian Cancer Screening Trial.^22^

We aimed to test the hypothesis that *AHRR* (cg05575921) hypomethylation is associated with the risk of smoking-related morbidity and mortality in the general population. Using the published lung cancer risk prediction model,^22^ the 6-year lung cancer risk was calculated: we then explored if *AHRR* (cg05575921) methylation extent improves lung cancer risk prediction among high-risk smokers, and thus helps to decide who should be offered lung CT scans.

## Materials and methods

### Study population

**Figure 1:**
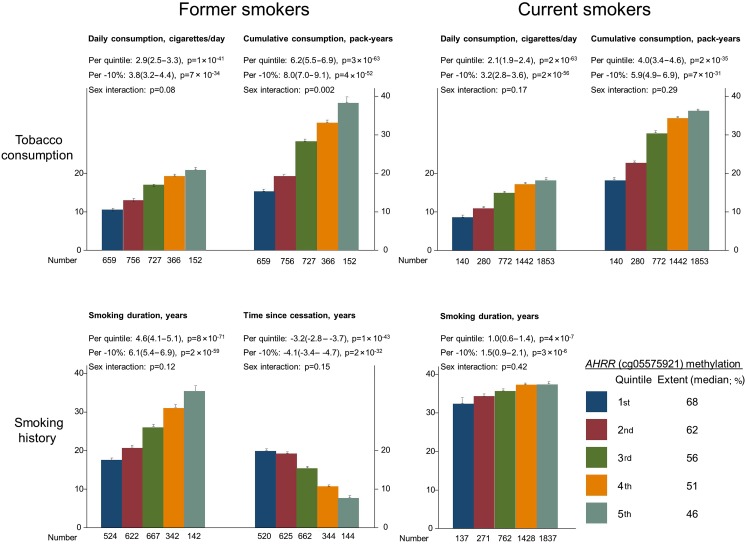
Smoking behaviour as a function of AHRR (cg05575921) methylation extent, among former and current smokers. The lower number of individuals, for smoking history compared with tobacco consumption, is explained by missing information on smoking history. Bar values are means and SEs. Estimates (95% CI) and p values per lower quintile and per 10% lower methylation extent were calculated with unadjusted linear regression. p Values for sex interaction were from a likelihood ratio test that compared the main effects in a linear regression model to a model also including a two-factor (sex by quintile of methylation extent) interaction term. AHRR, aryl hydrocarbon receptor repressor.

We studied 9234 consecutive individuals with available DNA from the 1991 to 1994 examination of the Copenhagen City Heart Study, a prospective study of the general population with equal emphasis on pulmonary and cardiac diseases (see flowchart in online supplementay figure 1)^23^ ^24^; 98% were whites of Danish origin. Copenhagen residents were invited to complete a questionnaire and undergo a physical examination. All participants gave written informed consent, and a Danish ethics committee approved the study (KF100.2039/91). Participants reported on smoking behaviour, alcohol consumption, occupational exposure to dust and/or welding fumes, exposure to passive smoking, education, and familial cases of lung cancer. The answers were reviewed together with an examiner at the day of attendance. Height and weight were measured, body mass index was calculated and blood samples were drawn for DNA.

10.1136/thoraxjnl-2016-208789.supp1 supplementay appendix

### Smoking behaviour

At the day of attendance in 1991–1994 when DNA was sampled, participants were asked whether they were current or former smokers. If they answered affirmative to either of these questions, they were asked about their current and former smoking behaviour, including age of smoking initiation, age of smoking cessation and number of daily consumed cigarettes, cheroots, cigars and weekly grams of pipe tobacco. Based on these answers, participants were categorised as never, former and current smokers; the two latter groups were defined as smokers. Also, pack-years corresponding to the consumption of 20 cigarettes or equivalent per day for 1 year was calculated for smokers. Thus, never, former and current smoking, quantitative smoking information and smoking history were all recorded only once, at the 1991–1994 examination.

### ***CHRNA3*** genotype

The rs1051730 genotype near the nicotinic acetylcholine receptor gene (*CHRNA3)* was determined with a Taqman assay as described earlier.^24^

### Methylation extent

The *AHRR* cg05575921 methylation extent was measured in duplicate samples of bisulphite treated DNA from peripheral blood drawn at the 1991–1994 examination (see online supplementary appendix). Measurements were adjusted for 13 batches, and the order of individuals examined, sampling and measurements were according to the day of the month for birthday (ie, the 1st–the 31st day of the month) of the individual, thus presumably random and non-differential to *AHRR* cg05575921 methylation extent, or other measured or unmeasured variables. Measurements were validated by Pyrosequencing as outlined in the online supplementary appendix.

### Morbidity and mortality

We selected COPD exacerbations, lung cancer and all-cause mortality a priori because we expected these to show the strongest association with a potential smoking-related variable. For COPD exacerbations (ICD8, codes 490-2 until 1993, and ICD10, J40-4 from 1994 and onwards), dates of hospital diagnoses with exacerbation were from the national Danish Patient Registry, from 1977 to April 2013, as done previously.^25^ For lung cancer (ICD7, codes 1624 or 4624 until 1977 and ICD10, code C34 from 1978 and onwards), date was from the national Danish Cancer Registry from 1943 to December 2012, as done previously.^24^ For death or emigration (n=110), information was from the national Danish Civil Registration System from date of examination until April 2013.

### Lung cancer risk prediction model

Since screening for lung cancer using CT scan reduces lung cancer mortality,^20^ risk prediction models to identify individuals most likely to benefit from lung cancer screening have been developed: the Prostate, Lung, Colorectal and Ovarian Cancer Screening Trial^22^ risk prediction model (PLCO_M2012_) was calculated as outlined in the online supplementary appendix.

### Statistical analysis

We used STATA V.13.1. All 9234 individuals were categorised into ranked quintiles of DNA *AHRR* (cg05575921) methylation extent, with the quintile with the highest methylation, presumably least exposed to smoking, as the reference group. We showed data in quintiles for simple illustrative presentation of the results; however, analyses of methylation extent on a continuous scale are also shown. We used linear regression, Cuzick's extension of the Wilcoxon rank-sum test and Pearson's χ^2^ test.

Follow-up time for each individual began at baseline, which was defined as date of the 1991–94 examination with DNA sampling, and ended at date of death, event, emigration, or December 2012 (lung cancer) or April 2013 (mortality), whichever came first. For COPD exacerbations, the model allowed for multiple events for the same individual, while taking into account that the failure-times within each individual could be correlated. Individuals with lung cancer prior to the date of examination were excluded from analyses of lung cancer. Minimum, median and maximum follow-up periods were 4 days, 19 years and 22 years for COPD exacerbation, 4 days, 19 years and 21 years for lung cancer and 4 days, 19 years and 22 years for all-cause mortality.

We plotted cumulative incidences, accounting for the competing risks of death and emigration, similarly,^26^ and tested differences between quintiles using Fine–Gray regression or log-rank trend tests. HRs for morbidity or all-cause mortality with 95% CIs were calculated using Cox proportional hazards regression. Time scale was chronological age taking into account that risk time began at date of the 1991–1994 examination with DNA sampling. This means that age is automatically adjusted for and is referred to as ‘age-adjusted’ as further described in the online supplementary appendix. Corresponding analyses using Fine–Gray regression with death and emigration as equal competing events were also conducted as sensitivity analyses. We applied two models adjusted for sex and age, and multifactorially additionally for the use of alcohol (continuous), body mass index (continuous), exposure to dust (no/yes), exposure to passive smoking (no/yes), history of cancer prior to attendance (no/yes), history of COPD exacerbations prior to attendance (no/yes; for lung cancer and all-cause mortality), familial history of lung cancer (no/yes), education level (categorical), smoking status (never, former current), current and cumulative consumption of tobacco. Covariates in the multifactorially adjusted models were included because they are used in the PLCO_M2012_ prediction model.^22^ For simplicity, we used the same model for all three endpoints. All covariates were recorded once, at the 1991–94 examination. We tested the proportional hazards assumption using Schoenfeld residuals and no major violations were observed. For further information on the statistical modelling, please see the online supplementary appendix.

We chose not to adjust p values for multiple comparisons. If we consider association analyses for two morbidity endpoints, and one mortality endpoint, Bonferroni correction for multiple comparisons would require p values <0.017 (0.05/3=0.017). However, as most p values reported are much below, adjustment for multiple comparisons would not affect the main conclusions of the present paper.

## Results

Median of *AHRR* (cg05575921) methylation extent among 9234 individuals was 56% (IQR, 50–63%). Ranked quintiles of *AHRR* (cg05575921) methylation extent were associated with all baseline characteristics at the time blood collection for DNA extraction, except personal history of cancer (table 1). To validate our Taqman assay, we also measured methylation extent in 170 individuals using Pyrosequencing at the *AHRR* (cg05575921) site, and the results from the two assays were strongly associated (R^2^=0.70, F=381, p<0.0001), comparable to other studies of two methylation measurement techniques.^27^ Overlap of morbidity and mortality, among all, and stratified by smoking status are given in online supplementary figure 2.

**Table 1:** Baseline characteristics of 9234 participants from the Copenhagen City Heart Study

		Leucocyte DNA methylation of cg05575921, quintiles					
	All	1st (highest)	2nd	3rd	4th	5th (lowest)	p Value*
Number	9234	1754	1783	1815	1862	2020	–
AHRR (cg05575921) methylation extent, %	56 (50–63)	68 (65–71)	62 (60–63)	56 (54–58)	51 (49–53)	46 (44–48)	–
Women, n (%)	5114 (55)	1196 (68)	1086 (61)	935 (52)	909 (49)	988 (49)	1×10^−45^
Age, years	60 (47–70)	59 (45–71)	62 (45–73)	62 (49–72)	60 (50–69)	59 (47–67)	0.0004
Education below 10 years, n (%)	5141 (56)	826 (47)	900 (50)	991 (55)	1158 (62)	1266 (63)	3×10^−30^
Body mass index, kg/m^2^	25 (22–28)	25 (23–28)	25 (23–28)	25 (23–28)	25 (22–28)	24 (22–27)	2×10^−18^
Registered COPD, n (%)	152 (1.7)	12 (0.7)	15 (0.8)	30 (1.7)	38 (2.0)	57 (2.8)	3×10^−7^
FEV_1_/FVC ratio <Lower limit of normal, n (%)	1445 (16)	106 (6)	116 (7)	261 (15)	440 (24)	522 (26)	4×10^−104^
Personal history of cancer, n (%)	458 (5)	89 (5)	97 (5)	86 (5)	94 (5)	92 (5)	0.76
Family history of lung cancer, n (%)	855 (9)	157 (9)	139 (8)	164 (9)	168 (9)	227 (11)	0.006
Ever smokers, n (%)	7120 (77)	796 (45)	1029 (58)	1493 (82)	1802 (97)	2000 (99)	<10^−300^
Alcohol, g/week	60 (12–156)	48 (12–108)	48 (12–120)	72 (12–156)	72 (24–180)	84 (24–192)	1×10^−34^
Occupational exposure to dust or fumes, n (%)	1713 (19)	211 (12)	239 (13)	314 (17)	438 (24)	511 (25)	1×10^−36^
Exposure to passive smoking, n (%)	3321 (36)	458 (26)	480 (27)	600 (33)	809 (44)	974 (48)	3×10^−68^
Predicted 6-year lung cancer risk†, %	0.69 (0.11–2.26)	0.05 (9×10^−4^–0.42)	0.16 (0.01–0.86)	0.55 (0.09–2.04)	1.14 (0.30–2.83)	1.26 (0.36–3.09)	<10^−300^

Values are median (IQR) for continuous variables and frequencies for categorical variables.

*p Values were calculated with Cuzick’s extension of the Wilcoxon rank-sum test (age, alcohol and predicted lung cancer risk) or Pearson's χ^2^-test (categorical variables).

†As defined in NEJM 2013;368:728–36. Smokers only.

### Smoking behaviour

The proportion of smokers was higher among men than among women (p=3×10^−39^, see online supplementary table S1), as expected. For both former and current smokers, ranked quintiles of lower cg05575921 methylation extent were associated with higher daily tobacco consumption (p=1×10^−41^ and 2×10^−63^), higher cumulative tobacco consumption (p=3×10^−63^ and 2×10^−35^), longer smoking duration (p=8×10^−71^ and p=4×10^−7^), and with shorter time since cessation (former smokers only: p=1×10^−43^); corresponding results for methylation extent on a continuous scale were similar (figure 1). Cumulative tobacco consumption in former smokers increased more steeply in men than in women across ranked quintiles of lower *AHRR* (cg05575921) methylation extent (p=0.002, interaction test). Median *AHRR* (cg05575921) methylation extent was 64% among never smokers, 60% among former smokers and 50% among current smokers (p<1×10^−300^, see online supplementary table S1). Among never smokers, *AHRR* (cg05575921) methylation extent was similar in those exposed to (n=512) and not exposed to (n=1575) passive smoking (65% and 64%, p=0.95).

The rs1051730 genotype near the nicotinic acetylcholine receptor gene (*CHRNA3*) is a likely causal genetic marker of the heaviness of smoking, with the T allele associated with increased consumption of tobacco.^24^ We, therefore, tested whether this genotype also was associated with the objectively measured *AHRR* (cg05575921) methylation extent: methylation extent declined by 0.67% (95% CI 0.39% to 0.97%) per T allele (p=5×10^−6^) among current smokers, and by 0.48% (0.18% to 0.78%, p=0.002) among all smokers (see online supplementary figure 3). These associations remained after adjustments for self-reported smoking, but was absent among never smokers (p=0.96, not shown).

### Morbidity

**Figure 2:**
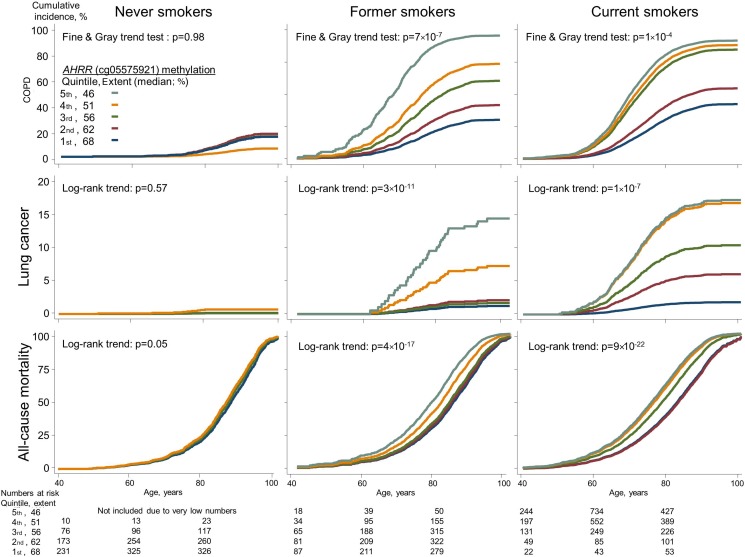
Cumulative incidences of COPD exacerbations, lung cancer and all-cause mortality as a function of ranked *AHRR* (cg05575921) methylation extent quintiles, among never, former and current smokers. Cumulative incidences were calculated by the Fine and Gray method, taking death and emigration as competing events into consideration, except for all-cause mortality for which only emigration was a competing event. For COPD exacerbations, the trend test is from the Fine and Gray regression instead of the log-rank trend test because the model allowed for multiple events for the same individual. Never smokers in 5th quintile were excluded due to very low numbers. AHRR, aryl hydrocarbon receptor repressor.

**Figure 3:**
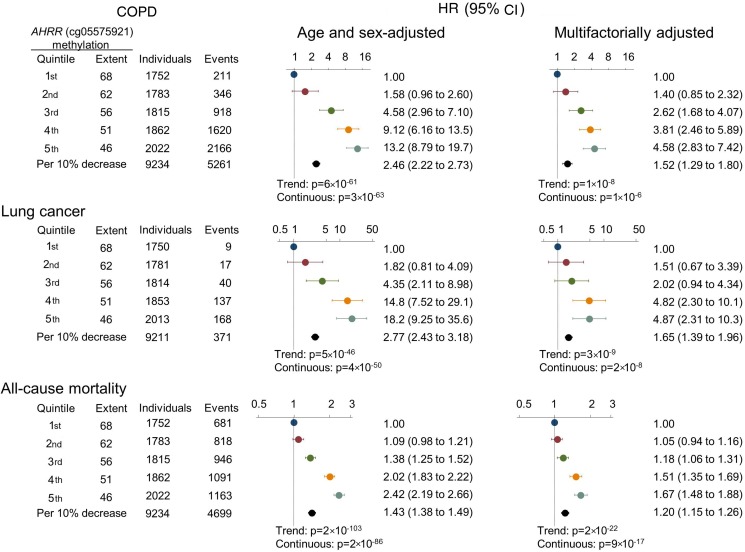
HRs of COPD exacerbation, lung cancer and all-cause mortality by *AHRR* (cg05575921) methylation extent quintiles and per 10% lower extent, among never, former and current smokers using a Cox regression model. Multifactorial adjustment was for age, sex, use of alcohol (continuous), body mass index (continuous), exposure to dust (no/yes), exposure to passive smoking (no/yes), history of cancer prior to attendance (no/yes), history of COPD prior to attendance (no/yes; for lung cancer and all-cause mortality), familial history of lung cancer (no/yes), education level (categorical), smoking status (never, former current), current and cumulative consumption of tobacco. p Values for trend were from the Cox regression model, where quintiles were inserted as a continuous variable. p Values (continuous) for the HR per 10% decrease of methylation extent are also shown. AHRR, aryl hydrocarbon receptor repressor.

For COPD exacerbation, we followed all individuals from time of examination, and during 143 000 person-years we detected 5261 events of COPD exacerbations among 1309 individuals. Among former and current smokers, there was an association between ranked quintiles of lower *AHRR* (cg05575921) methylation extent with higher incidence of COPD exacerbation (p=7×10^−7^ and p=1×10^−4^, Fine and Gray trend test) (figure 2, upper panels). The HRs for COPD exacerbation for individuals in the 5th versus 1st quintile of methylation extent among never, former and current smokers combined were 13.2 (95% CI 8.79 to 19.7) in the age and sex adjusted model, and 4.58 (2.83 to 7.42) in the multifactorially adjusted model (figure 3).

For lung cancer, we followed 9211 individuals without lung cancer at the time of examination, and during 141 000 person-years we detected 352 individuals with lung cancer. Among former and current smokers, there was an association across ranked quintiles of lower *AHRR* (cg05575921) methylation extent with higher incidence of lung cancer (p=3×10^−11^ and p=1×10^−7^, log-rank trend test), whereas this was not seen among never smokers (figure 2 middle panels). The HRs for lung cancer for individuals in the 5th versus 1st quintile of methylation extent among never, former and current smokers combined were 18.2 (95% CI 9.25 to 35.6) in the age and sex adjusted model, and 4.87 (2.31 to 10.3) in the multifactorially adjusted model (figure 3). Risk estimates were slightly attenuated when taking competing risk of death and emigration into consideration (compare figure 3 with online supplementary figure 4).

### Mortality

For all-cause mortality, 4396 individuals died during 143 000 person-years. Among former and current smokers, there was an association across ranked quintiles of lower *AHRR* (cg05575921) methylation extent with higher all-cause mortality (p=4×10^−17^ and p=9×10^−22^, log-rank trend test). In current smokers, the difference of median age at death between individuals in the 1st and 5th methylation extent quintile was 8.5 years (not shown) (figure 2 lower panels). The HRs for all-cause mortality for individuals in the 5th versus 1st quintile of methylation extent among never, former and current smokers combined were 2.42 (95% CI 2.19 to 2.66) in the age and sex adjusted model, and 1.67 (1.48 to 1.88) in the multifactorially adjusted model (figure 3).

### Prediction of lung cancer risk

**Figure 4:**
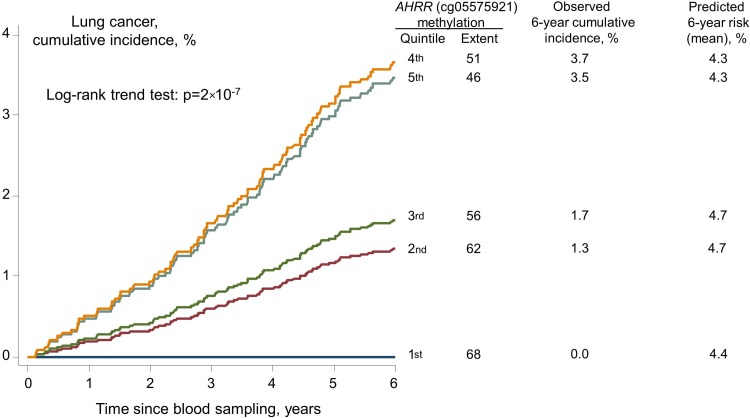
Cumulative incidence of lung cancer among 2576 high-risk smokers, and observed and predicted 6-year risk by *AHRR* (cg05575921) methylation extent quintiles. High risk was defined as predicted PLCO_M2012_ 6-year risk>1.3455%.^22^ AHRR, aryl hydrocarbon receptor repressor; PLCO_M2012_, Prostate, Lung, Colorectal and Ovarian Cancer Screening Trial.

Among high-risk smokers (n=2576) eligible for lung cancer screening by CT scan due to a PLCO_M2012_^22^ lung cancer 6-year risk above 1.3455% (see online supplementary figure 5), the observed cumulative incidence of lung cancer after 6 years for individuals in the 5th and 1st quintile of methylation extent were 3.7% and 0.0% (p=2×10^−7^, log-rank trend test across all quintiles, figure 4), whereas the predicted PLCO_M2012_ lung cancer 6-year risks were similar (4.3% and 4.4%, p=0.77 trend test). The 6-year follow-up was chosen, since it was used in the publication where the prediction model was developed.^22^ After more than 20 years of follow-up, no smoker in the highest quintile of *AHRR* (cg05575921) methylation extent developed lung cancer (see online supplementary figure 6); however, this group had a relatively low number of individuals. Prediction of lung cancer risk with receiver operating characteristic (ROC) statistics for *AHRR* (cg05575921) methylation extent and the PLCO_M2012_ risk estimate produced similar area under curve (AUC) estimates in all 7120 ever smokers (p=0.11 for difference), but an AUC of 61% for *AHRR* (cg05575921) methylation extent versus 51% for the PLCO_M2012_ risk estimate in 2576 high-risk smokers (p=0.0001 for difference) (see online supplementary figure 7).

## Discussion

Among 9234 individuals from the general population, *AHRR* (cg05575921) methylation extent was lower among smokers than non-smokers, and inversely correlated with smoking intensity, in accordance with previous findings.^9–15^ ^28^ *AHRR* (cg05575921) hypomethylation was predictive of high risk of COPD exacerbation, lung cancer and all-cause mortality, also after adjustment for past and current smoking behaviour. Within a large group of smokers with a 6-year a priori risk of lung cancer above the current cut-off for lung CT scan, *AHRR* cg05575921 methylation extent separated individuals into a very low, an intermediate and a high-risk group. This is a novel finding.

*AHRR* (cg05575921) methylation extent was an accurate marker of several components of the smoking history. *AHRR* expression is induced by polycyclic aromatic hydrocarbons^29^ and cg05575921 methylation marks an upregulation of the expression of *AHRR* in blood cells^30^ and lung tissue.^12^ Whether cg05575921 hypomethylation of leucocyte DNA in itself has any direct causal role in the development of smoking-related diseases is not known. Observational studies such as ours cannot clarify this, but the AHRR protein is located in the cell nucleus,^29^ and silencing of AHRR enhances tumour growth pleiotropically through increased Aryl Hydrocarbon Receptor activity.^31^

Cg05575921 methylation extent varied with smoking behaviour, even among former smokers, who had quit many years before blood sampling, which leads to different exposure to toxic effects of smoking. This suggests that *AHRR* cg05575921 methylation extent is less dynamic in adults than in new-borns, where more rapid attenuation of hypomethylation is seen once the antenatal exposure is removed.^32^ A small number of never smokers have low *AHRR* (cg05575921) methylation extent, presumably due to environmental exposures and may have increased risk of lung cancer.

Three observations support the notion that measurement of *AHRR* (cg05575921) methylation extent is superior to self-reported smoking behaviour when assessing an individual's risk of smoking-related morbidity and mortality. First, *AHRR* (cg05575921) methylation extent was associated with the rs1051730 genotype of the nicotinic acetylcholine receptor gene (*CHRNA3*), a variant associated with increased smoking behaviour,^6^ also after adjustments for self-reported smoking. This suggests that *AHRR* (cg05575921) methylation extent is superior at capturing individuals' ‘true’ smoking behaviour. Second, risk estimates of future smoking-related morbidity by methylation extent remained high even after adjustments for self-reported smoking. Third, about 70% of the lung cancer burden is attributed to smoking alone^1^ and in ROC statistics of incident lung cancer in all smokers, *AHRR* (cg05575921) methylation extent performed similarly to the PLCO_M2012_ prediction model, which includes information on present and former smoking behaviour. Among high-risk smokers, the relatively bad performance of PLCO_M2012_ prediction model might be due to the fact that it was developed to produce a binary risk cut-off for lung CT scan in smokers and not to be used as a continuous risk variable.

If *AHRR* (cg05575921) methylation extent is indeed a better indicator of the harmful effects of smoking than self-reported smoking behaviour, this points towards the potential clinical use of *AHRR* (cg05575921) methylation extent in stratifying smokers into different risk groups prior to lung CT scan. For this purpose, the relatively stable *AHRR* (cg05575921) methylation extent could in the future be supplemented with short-term objective epigenetic markers of smoking behaviour. It could seem risky to base a decision on lung CT scan on a single blood test sampled years previously, and linked to smoking at the time of sampling, to predict the individual's lung cancer risk. Similar arguments are also valid for the PLCO_M2012_ prediction model used to select for lung CT scan. While *AHRR* (cg05575921) methylation extent and the PLCO_M2012_ prediction model only predict what will happen for the average person, predictions are naturally not exact for a specific individual.

Strengths of this study include the large number of general population individuals followed for more than 20 years without losses to follow-up. The Danish health registers allowed us to examine associations with three endpoints and this combined with the high frequency and heaviness of tobacco smoking in Denmark in the 1990s gave us adequate power to test the hypotheses.

Some limitations must, however, be acknowledged. This is the first large-scale application of a simple assay quantifying DNA methylation; however, two arguments support the notion that the assay captured relevant information. First, the measurements correlated with results from Pyrosequencing. Second, we could reproduce the expected association with the smoking-related *CHRN3A* genotype.

Also, COPD exacerbation during follow-up was only defined by diagnostic codes for hospital admissions, and not additionally by glucocorticosteroide and/or antibiotic treatment. We also lacked information on other variables, which may impact methylation, for example exposure to benzene, lead and arsenic.^33^ An invalid, non-differential measurement technique would only tend to pull associations with phenotypes, morbidity and mortality towards the null hypothesis, and thus cannot explain our positive findings.

Misclassification of diagnoses from the Danish registries is a potential limitation, but is likely non-differential by methylation extent and can therefore not explain our results on COPD. Importantly, for lung cancer, diagnoses are based on histological confirmation by a fully trained pathologist and registration is compulsory by law. For all-cause mortality, there is no misclassification in Denmark.

The 9234 individuals examined only represent 56% of the invited population. Thus, smoking-related death or severe disease may have prevented some from being examined. However, this potential self-selection of healthy individuals cannot explain our positive findings of associations with self-reported smoking, morbidity and mortality. Importantly, we detected the presumed unbiased association with the genotype of the smoking-related *CHRN3A* genotype, for example the genotype distributions among ever and never smokers were similar (p=0.24, χ^2^ test; data not shown), suggesting that if present, selection might be of minor importance. Furthermore, we only measured *AHRR* (cg05575921) methylation extent once, precluding adjustment for within-person variation, regression dilution bias and determination of the natural pattern of methylation after smoking cessation. Nevertheless, we did detect an association of ranked *AHRR* (cg05575921) methylation quintiles with time since cessation.

It could be argued that independent validation of our findings would be preferable; however, the strengths and magnitudes of the associations, and the biological plausibility involving a well-known disease causing lifestyle, argue against the possibility that the findings are due to chance alone.

In conclusion, we found that *AHRR* (cg05575921) hypomethylation was associated with smoking, high smoking-related morbidity and with high all-cause mortality. Among smokers at high risk of lung cancer, and eligible to screening with CT scan, *AHRR* (cg05575921) methylation extent could help to identify a subgroup with the potential most benefit from a lung CT scan. Cost–benefit analyses of adding *AHRR* (cg05575921) methylation extent to selection for lung CT scan should be explored in future studies.
