# Supplementary material for: AHRR (cg05575921) hypomethylation marks smoking behaviour, morbidity and mortality
Source: Thorax. 2017 Jan 18;72(7):646–53. doi: 10.1136/thoraxjnl-2016-208789 (PMC5520281; doi:10.1136/thoraxjnl-2016-208789)
Supplement: supplementay appendix [file thoraxjnl-2016-208789supp001.pdf]

AHRR (cg05575921) hypomethylation marks smoking behavior, morbidity and mortality

Supplementary Appendix

Stig E Bojesen 1,2,3, Nicholas Timpson 4, Caroline Relton 4, George Davey Smith 4, Børge G Nordestgaard 1,2,3

1 Department of Clinical Biochemistry, Herlev and Gentofte Hospital, Copenhagen University Hospital, Denmark

2 Faculty of Health and Medical Sciences, University of Copenhagen, Denmark

3 The Copenhagen City Heart Study, Frederiksberg Hospital, Copenhagen University Hospital, Denmark

4 MRC Integrative Epidemiology Unit (IEU), School of Social and Community Medicine, University of Bristol, UK

Table of contents Page No.

Measurement of lung function 2
Description of the *AHRR* (cg05575921) methylation measurement technique 2-4
Validation using Pyrosequencing 4-5
Statistical Modeling 5-6
Supplementary Table 1 7
Supplementary Figure 1 8
Supplementary Figure 2 9
Supplementary Figure 3 10
Supplementary Figure 4 11
Supplementary Figure 5 12
Supplementary Figure 6 13
Supplementary Figure 7 14
References 15

Lung function

A dry wedge spirometer (Vitalograph; Maids Moreton, Buckinghamshire, UK) calibrated daily with a 1-liter syringe was used. The highest out of three measurements of forced expiratory volume in the first second (FEV_1) and the forced vital capacity (FVC) in liters were used.[1] Lower limit of normal was calculated as the difference between the predicted value and 1.645 times the standard error of the estimate for measured values of FEV_1/FVC ratio separately for men and women.

DNA

DNA was isolated from frozen whole blood samples using the Qiagen Blood Kit resulting in an eluate with 5-30ng/microliter. Twenty microliter of this was treated with bisulfite using the EZ-96 DNA Methylation Gold kit (Zymo Research). Elution volume was 60 microliter. The laboratory technicians were blinded to smoking and disease status of the individuals.

PCR assay

We measured DNA *AHRR* cg05575921 methylation extent using a Taqman assay developed in our own laboratory: the bisulfite treated DNA was amplified using forward and reverse PCR primers, which were designed to bind to DNA around the cg05575921 site on sequences without genetic or possible CpG methylation variation. The probes detected either the unmethylated, and therefore conversed T residue, or the methylated and therefore conserved C residue.

**Name**	**Sequence 5’->3’**
Forward primer	GGGATTGTTTATTTTTGAGAGGGTAGTTT
Reverse primer	CTACCAAAACCACTCCCAAAACC
Probe detecting unmethylated cg05575921	VIC-AACCCAACC**A**AATACA
Probe detecting methylated cg05575921	FAM-AACCCAACC**G**AATACA

The thermal cycling profile was a conventional Taqman profile with 10 minutes at 95°C followed by 50 cycles of 15 seconds at 94°C, 60 seconds at 60°C, and 72˚C for 15 seconds. There was a final extension step of 72˚C for 5 minutes before amplicons were cooled to 4˚C.

On each 384-well plate, DNA from participants was examined in duplicates. Duplicate samples for a standard curve were included in each plate. These 11 samples were pipetted in steps of 10% with decreasing amounts of EpiTect control DNA, methylated (Qiagen: kat.no. 59655), from 100% to 0% mixed with increasing amounts of EpiTect control DNA, unmethylated (Qiagen: kat.no. 59665), from 0% to 100%. All standard curve samples were diluted to a concentration of 5ng/microL, and 1 microL was used for each well. Also, we included duplicates of the same internal control sample across all plates. In total, the samples were measured in 13 batches, and when a new batch of the stock samples for the standard curves were used, the last plate using the old standard curve samples were also run with the new standard curve samples, and adjusted for batch effects, ensuring that all samples were essentially measured with the same calibration. The order of individuals examined, sampling and measurements were according to the day of the month for birthday (ie. the 1st to the 31st day of the month) of the individual and thereby non-differential to risk factors in Table 1.

After the end of the PCR reaction, the plates were read on a Viaa 7 Real-Time PCR System (Life Technologies), the raw signal intensities from the two probes exported to excel files, and the calculations were done using STATA. Samples were failed if the methylation extent from the duplicates were more than 30% from each other.

Failed samples were attempted measured again. Therefore, valid measurements of methylation extent were available for more than 99.8% of available DNA samples.

Assay precision

Each 384-well plate contained two identical internal control samples. Plates failed if the methylation percentages from these were more than 30 relative percent discrepant, eg. a second measurement below 35%, or above 65% after a first measurement of 50%. For the accepted duplicate measurements, correlation between the first and second measurement was good (R^2=0.88), and even better for individuals with hypomethylation. Imprecision was measured across plates with the use of the result of internal control samples. Mean, standard deviation, and coefficient of variation were calculated. Coefficients of variation at 71% methylation extent varied from 5.0 to 6.7% for different stocks of the internal control.

Validation using Pyrosequencing

Validation of the the TaqMan measurement was done by Pyrosequencing in 170 randomly selected samples to be able to detect a 5% difference between the two measurements with alpha=0.05, and statistical power of 80%. Five hundred ng genomic DNA was bisulphite modified according to manufacturers recommended conditions (Zymo, Irvine, CA, USA). Pyrosequencing using a PyroMark ID pyrosequencer (Qiagen, Manchester, UK) was carried out, also according to the manufacturer's recommendations. Specifically, approximately 200 ng bisulphite converted DNA was added to a 50 microL PCR reaction containing 1x Hot star Taq mastermix (Qiagen, Manchester, UK), 0.4 micromoles/L of each oligonucleotide and dH2O. PCR amplification was 95˚C for 15 minutes followed by 50 cycles of 95˚C for 15 seconds, 54˚C for 30 seconds, and 72˚C for 15 seconds. There was a final extension step of 72˚C for 5 minutes before amplicons were cooled to 4˚C. Forty microL of amplicons were utilised for downstream single strand preparation and hybridisation of 0.4 micromoles/L sequencing primer using a Qiagen vacuum prep tool and workstation according to manufacturer’s instructions.

Pyrosequencing assays and primers were designed using the PyroMark Assay Design Software 2.3 (Qiagen, Manchester, UK).

Oligo details were as follows:

**Name**	**Sequence 5'->3'**
Forward oligo	GGATTGTTTATTTTTGAGAGGGTAGT
Reverse oligo	Biosg-AAAAAAACCCTACCAAAACCACTC
Sequencing oligo	GGTTTTGGTTTTGTTTTGTA

CpGs captured: 1. Annealing temp 54˚C, genomic location: Chr5:373,320-373,463, amplicon length:144.

Statistical Modeling

This is a prospective cohort study of time-to-event, for which individuals from the general population were questioned, examined and had their blood drawn at one day during 1991 through 1994. The individual information obtained here was used as covariates to model risk of events after this baseline (=examination) date. Instead of using time since baseline date as time scale, we used time since birth in years (=chronological age), while truncating time before baseline date, the so-called left-truncated age. Cox proportional hazard model was the default time-to-event model and the Fine and Gray proportional subdistribution hazard model was employed in sensivtity analyses (Suppl. Figure 4), and in drawing survival curves (Figure 2, 4, and Suppl. Figure 6). This was done because the Fine and Gray modelling takes the competing risk of death or emigration into account. This is important because *AHRR* cg05575921 hypomethylation was associated with higher mortality, and risk of death and emigration therefore compete with risks of COPD exacerbations and lung cancer. This can produce inflated risk estimates in Cox compared with Fine and Gray models.

The Prostate, Lung, Colorectal, and Ovarian Cancer Screening ($PLCO_{M2012}$) risk prediction model was specifically developed and validated to predict risk of lung cancer, which is highly associated with tobacco consumption. Because *AHRR* cg05575921 hypomethylation was associated with high tobacco consumption, we included the variables in the $PLCO_{M2012}$ risk prediction model as covariates without modifications in our multifactorially adjusted model. To simplify the presentation, the same model was also used for risk of COPD exacerbation and all-cause mortality. The results from the simple age- and sex-adjusted model were presented in order to facilitate comparability with other studies, which might not have records of all covariates in the multifactorially adjusted model. We also adopted the 6-year follow-up time reported in $PLCO_{M2012}$[2] in Figure 4. We thus calculated risk for individuals as described in [2], using age, education level (questionnaire), body mass index (measured), presence of COPD (register data), personal history of cancer (register data), family history of lung cancer (questionnaire), smoking status (questionnaire), smoking intensity (questionnaire), smoking duration (questionnaire), smoking cessation time (questionnaire), and the $PLCO_{M2012}$ model constant. The lung cancer 6-year cut-off in this model was 1.3455%, and individuals above were classified as high-risk smokers eligible for a CT scan.

In analyses of smoking behavior, we tested for interaction between sex and *AHRR* cg05575921 methylation extent quintile by using a likelihood ratio test that compared the main effects in a linear regression model to a model also including a two-factor (sex by methylation extent quintile) interaction term.

Suppl Table 1: Baseline characteristics of 9234 participants from the Copenhagen City Heart Study.

		Smoking status			
	**All**	Never smokers	Former smokers	Current smokers	P-value*
**Number**	9234	2087	2660	4487	-
***AHRR* (cg05575921) methylation, %**	56 (50-63)	64 (60-68)	60 (55-65)	50 (46-54)	$<1*10^{-300}$
**Women, n (%)**	5114 (55)	1421 (68)	1353 (51)	2340 (52)	$3*10^{-39}$
**Age, years**	60 (47-70)	56 (40-70)	65 (53-73)	59 (47-68)	0.36
**Education below 10 years, n (%)**	5141 (56)	1000 (48)	1446 (54)	2695 (60)	$3*10^{-30}$
**Body mass index, kg/m^2**	25 (22-28)	25 (22-28)	26 (23-29)	24 (22-27)	$2*10^{-15}$
**Registered chronic obstructive pulmonary disease, n (%)**	152 (1.7)	7 (0.3)	57 (2.1)	88 (2.0)	$5*10^{-7}$
**FEV1/FVC ratio < Lower limit of normal, n (%)**	1445 (16)	103 (5)	344 (13)	998 (23)	$7*10^{-76}$
**Personal history of cancer, n (%)**	458 (5)	87 (4)	161 (6)	210 (5)	0.006
**Family history of lung cancer, n (%)**	855 (9)	181 (9)	248 (9)	426 (9)	0.56
**Alcohol, g/week**	60 (12-156)	36 (0-96)	72 (12-144)	84 (24-180)	$8*10^{-56}$
**Occupational exposure to dust or fumes, n (%)**	1713 (19)	232 (11)	487 (18)	994 (22)	$1*10^{-25}$
**Exposure to passive smoking, n (%)**	3321 (36)	512 (25)	674 (25)	2135 (48)	$7*10^{-112}$
**Predicted 6-year lung cancer risk§, %**	0.69 (0.11-2.26)	-	0.22 (0.02-1.10)	1.10 (0.24-2.92)	$<1*10^{-300}$

Values are median (interquantile range) for continous variables and frequencies for categorical variables.

* P-values were calculated with Cuzick's extension of the Wilcoxon rank-sum test (cg05575921 methylation, age, alcohol, and predicted lung cancer risk), or Pearson's Chi2-test (categorical variables).

§ As defined in NEJM 2013;368:728-36. Smokers only.

Suppl. Figure 1
Flowchart of the study individuals from the Copenhagen City Heart Study, 1991-94 examination.

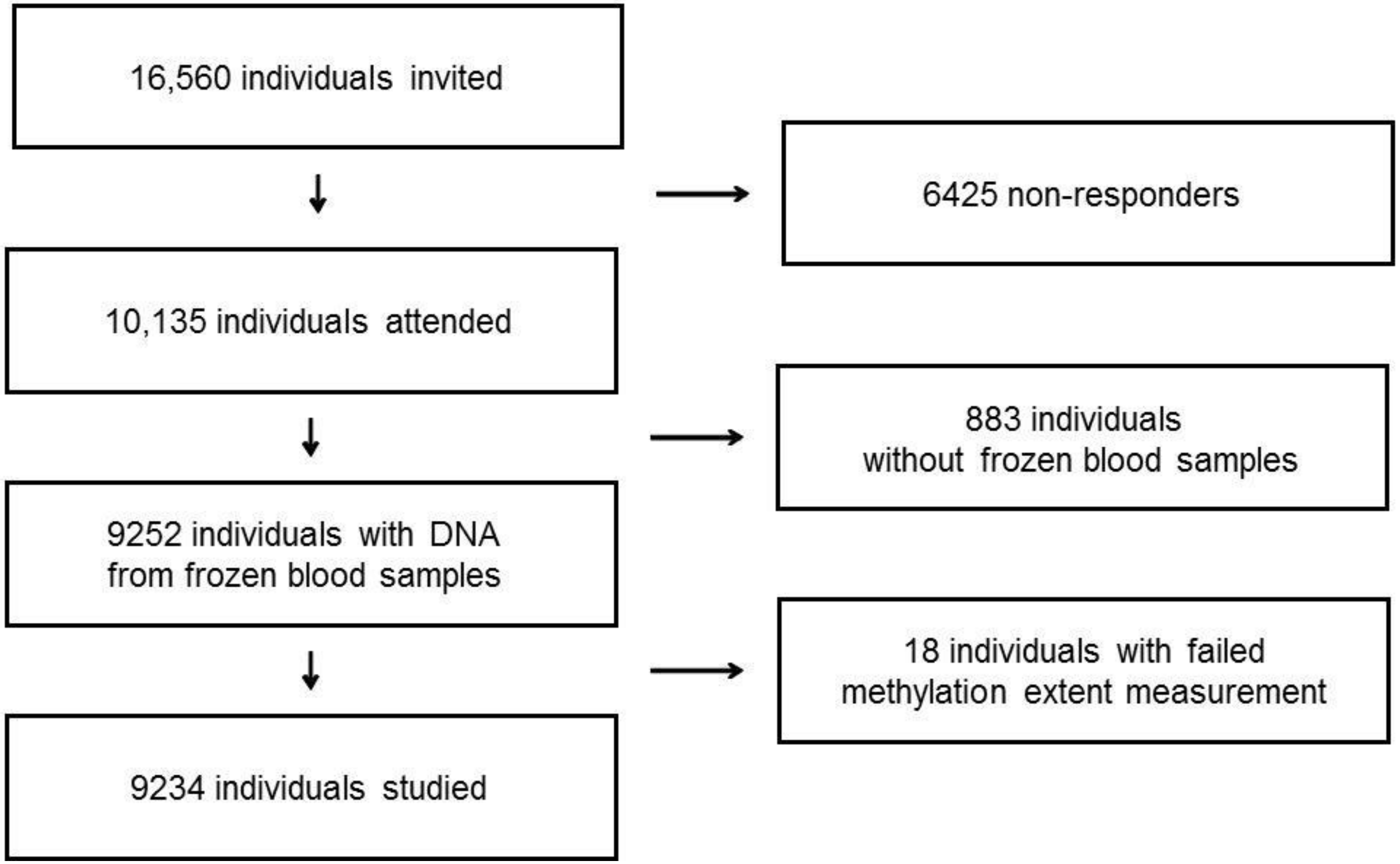

Suppl. Figure 2
Overlap of morbidity and mortality for individuals at the end of follow-up, among all and stratified by smoking status. Each individual can only have one event. Therefore the number for chronic obstructive pulmonary disease exacerbation is much lower than shown in Figure 3. Individuals with lung cancer at the day of attendance are included. Therefore the number for lung cancer are slightly higher than shown in Figure 3.

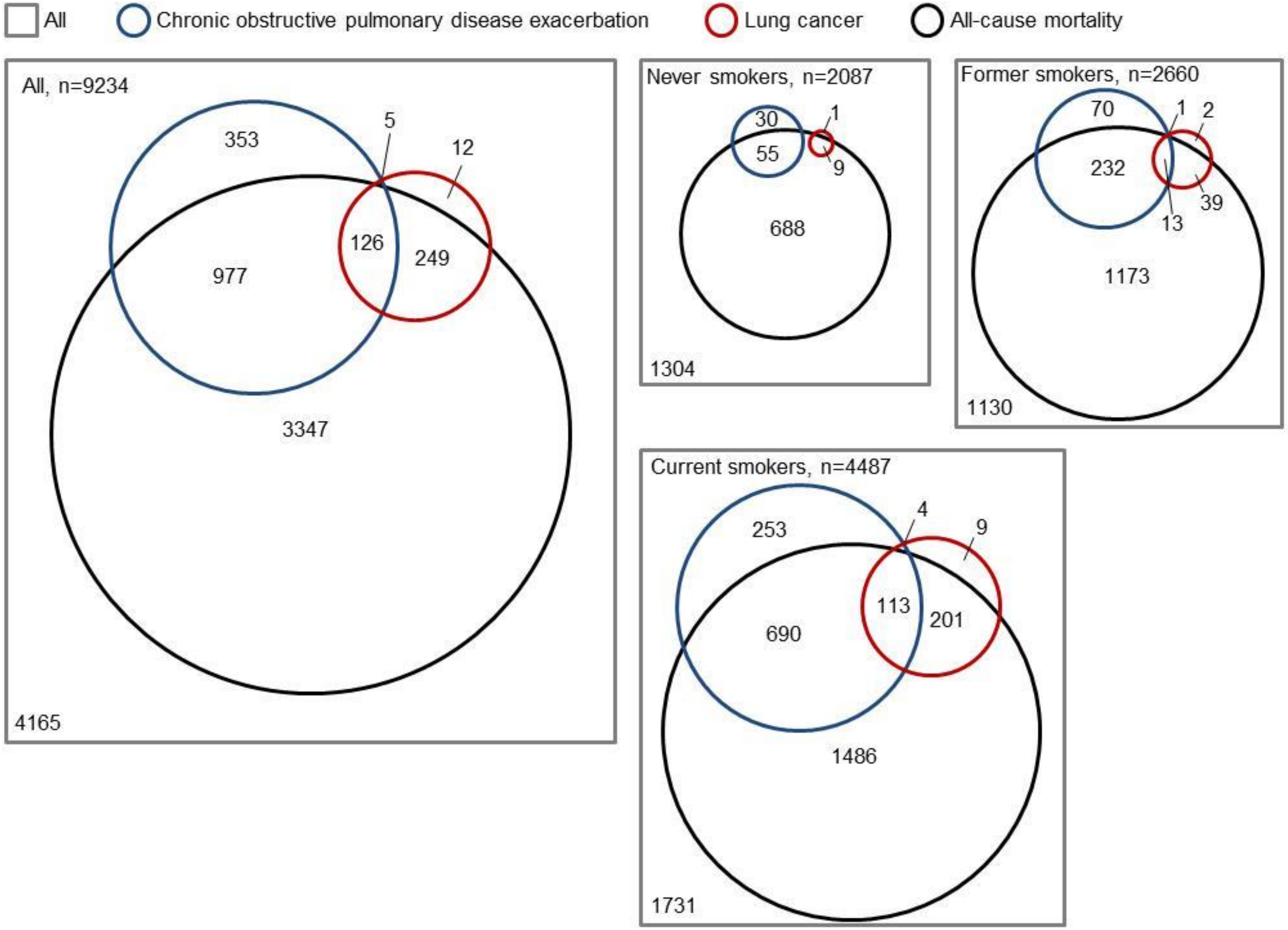

Suppl. Figure 3
Rs1051730 genotype near the nicotinic acetylcholine receptor (*CHRNA3)* and *AHRR* (cg05575921) methylation extent (mean, standard error) among current and all smokers. For never smokers (n=2086), p=0.96 (not shown). For 40 individuals genotype was not available. CI=confidence interval.

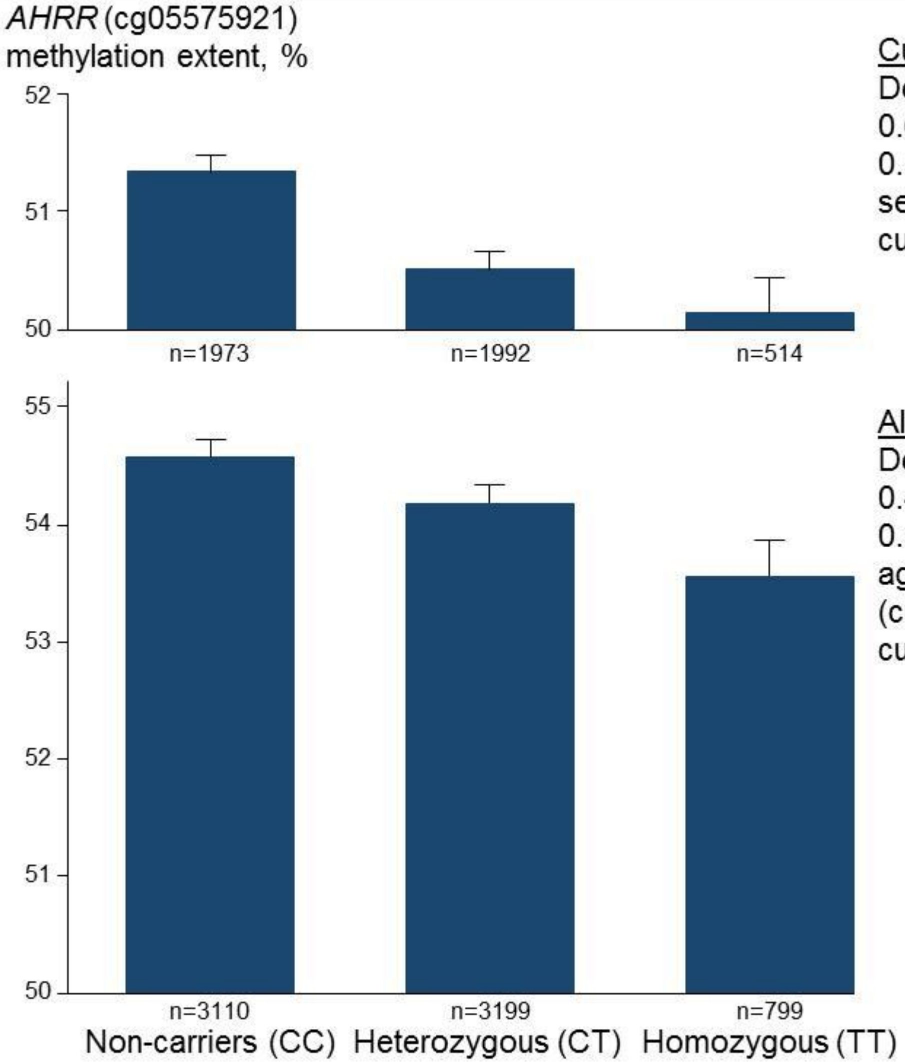

Current smokers (n=4479)
Decline per T-allele:
0.67% (95%CI: 0.39-0.97, $p=5*10^{-6}$, unadjusted)
0.51% (95% CI: 0.24-0.79, p=0.0003, adjusted for sex, age, smoking duration, and current and cumulative tobacco consumption)

All smokers (n=7108)
Decline per T-allele:
0.48% (95%CI: 0.18-0.78, p=0.002, unadjusted)
0.36% (95%CI: 0.12-0.60, p=0.003, adjusted for sex, age, smoking duration, time since smoking cessation (current smokers=0), and cumulative and current/former tobacco consumption)

Suppl. Figure 4
Subhazard ratios of chronic obstructive pulmonary disease exacerbation and lung cancer by AHRR (cg05575921) methylation extent quintiles and per 10% decrease, among never, former, and current smokers using a Fine and Gray competing risk regression model. Death and emigration were competing events. Therefore, estimates for all-cause mortality is not shown. Multifactorial adjustment was for age, sex, use of alcohol (continuous), body mass index (continuous), exposure to dust (no/yes), exposure to passive smoking (no/yes), history of cancer prior to attendance (no/yes), history of chronic obstructive pulmonary disease prior to attendance (no/yes; for lung cancer and all-cause mortality), familial history of lung cancer (no/yes), education level (categorical), smoking status (never, former current), current and cumulative consumption of tobacco. The model allowed for multiple COPD exacerbations per individual, while taking into account that the failure-times within each individual could be correlated. P-values for trend were from the Fine and Gray competing risk regression model, where quintiles were inserted as a continuous variable. P-values (continuous) for the subhazard ratios per 10% decrease of methylation extent are also shown.

Chronic obstructive pulmonary disease

Subhazard ratio (95% confidence interval)

AHRR (cg05575921) methylation Quintile	Extent	Individuals	Events	Age and sex-adjusted	Multifactorially adjusted
1st	68%	1752	211	1.00	1.00
2nd	62%	1783	346	1.60 (0.97 to 2.63)	1.39 (0.84 to 2.30)
3rd	56%	1815	918	4.22 (2.72 to 6.53)	2.38 (1.52 to 3.71)
4th	51%	1862	1620	7.04 (4.75 to 10.4)	3.04 (1.94 to 5.76)
5th	46%	2022	2166	9.30 (6.21 to 13.9)	3.48 (2.12 to 5.73)
Per 10% decrease		9234	5261	2.08 (1.88 to 2.31)	1.36 (1.14 to 1.62)

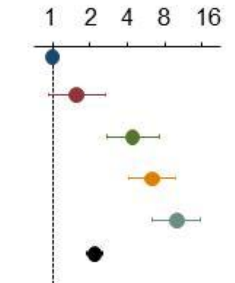

Trend: $p=8*10^{-44}$
Continuous: $p=1*10^{-44}$

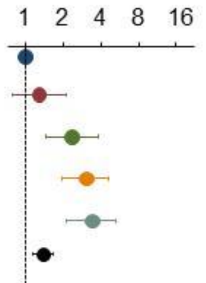

Trend: $p=3*10^{-5}$
Continuous: $p=6*10^{-4}$

Lung cancer

Quintile	Extent	Individuals	Events	Age and sex-adjusted	Multifactorially adjusted
1st	68%	1750	9	1.00	1.00
2nd	62%	1781	17	1.86 (0.83 to 4.16)	1.55 (0.70 to 3.44)
3rd	56%	1814	40	4.02 (1.95 to 8.28)	1.85 (0.90 to 3.80)
4th	51%	1853	137	12.0 (6.12 to 23.6)	4.00 (1.96 to 8.16)
5th	46%	2013	168	13.5 (6.92 to 26.5)	3.77 (1.83 to 7.79)
Per 10% decrease		9211	371	2.40 (2.14 to 2.68)	1.47 (1.25 to 1.73)

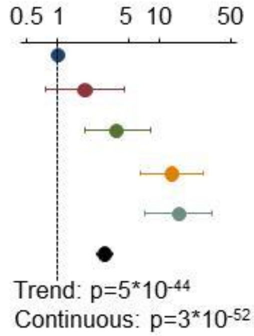

Trend: $p=5*10^{-44}$
Continuous: $p=3*10^{-52}$

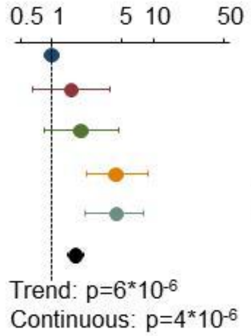

Trend: $p=6*10^{-6}$
Continuous: $p=4*10^{-6}$

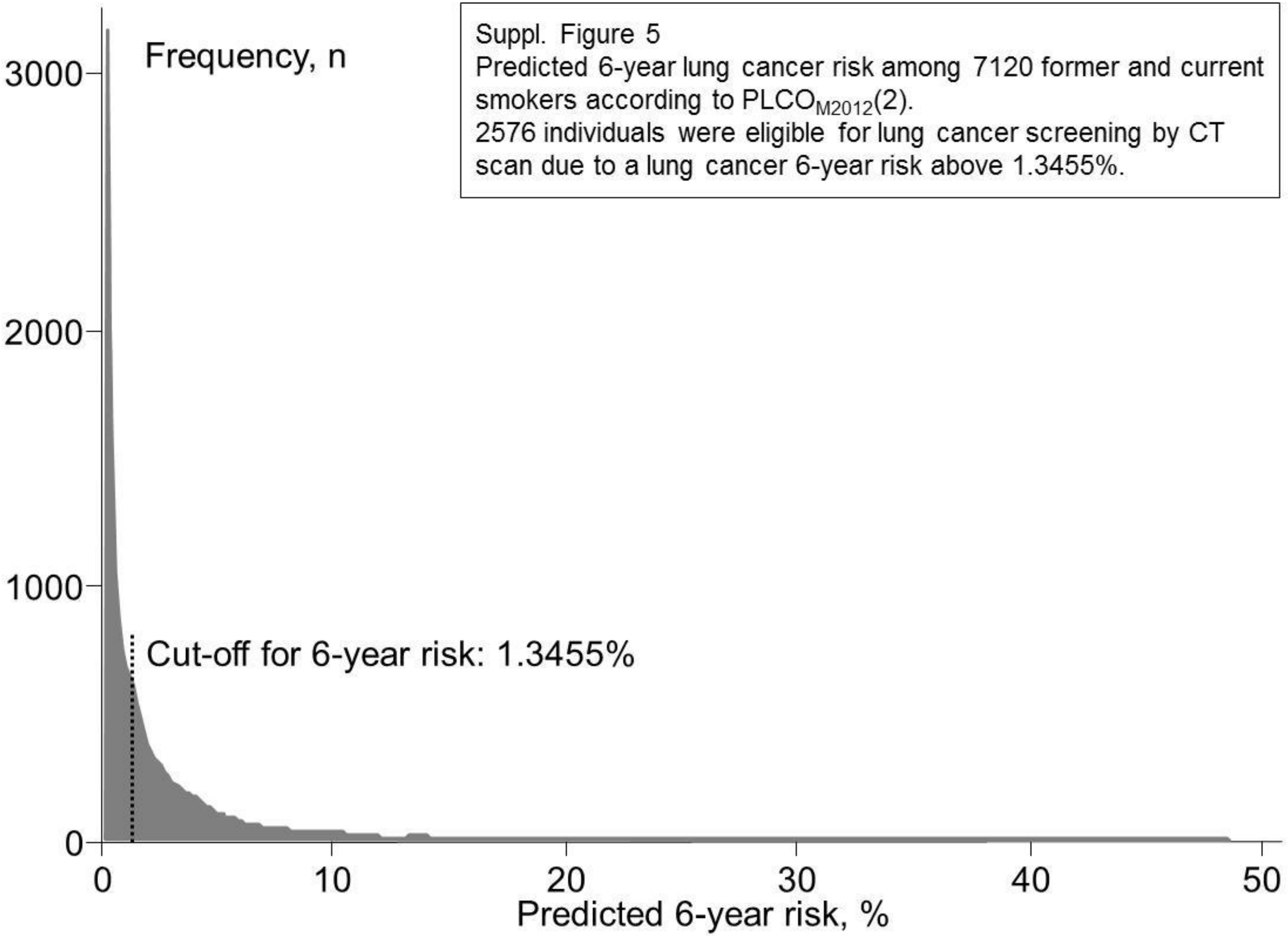

Suppl. Figure 5
Predicted 6-year lung cancer risk among 7120 former and current smokers according to $PLCO_{M2012}$(2).
2576 individuals were eligible for lung cancer screening by CT scan due to a lung cancer 6-year risk above 1.3455%.

Suppl. Figure 6
Cumulative incidence of lung cancer among 2576 high-risk smokers by *AHRR* (cg05575921) methylation extent quintiles. High risk was defined as predicted $PLCO_{M2012}$ 6-year risk>1.3455%(2).

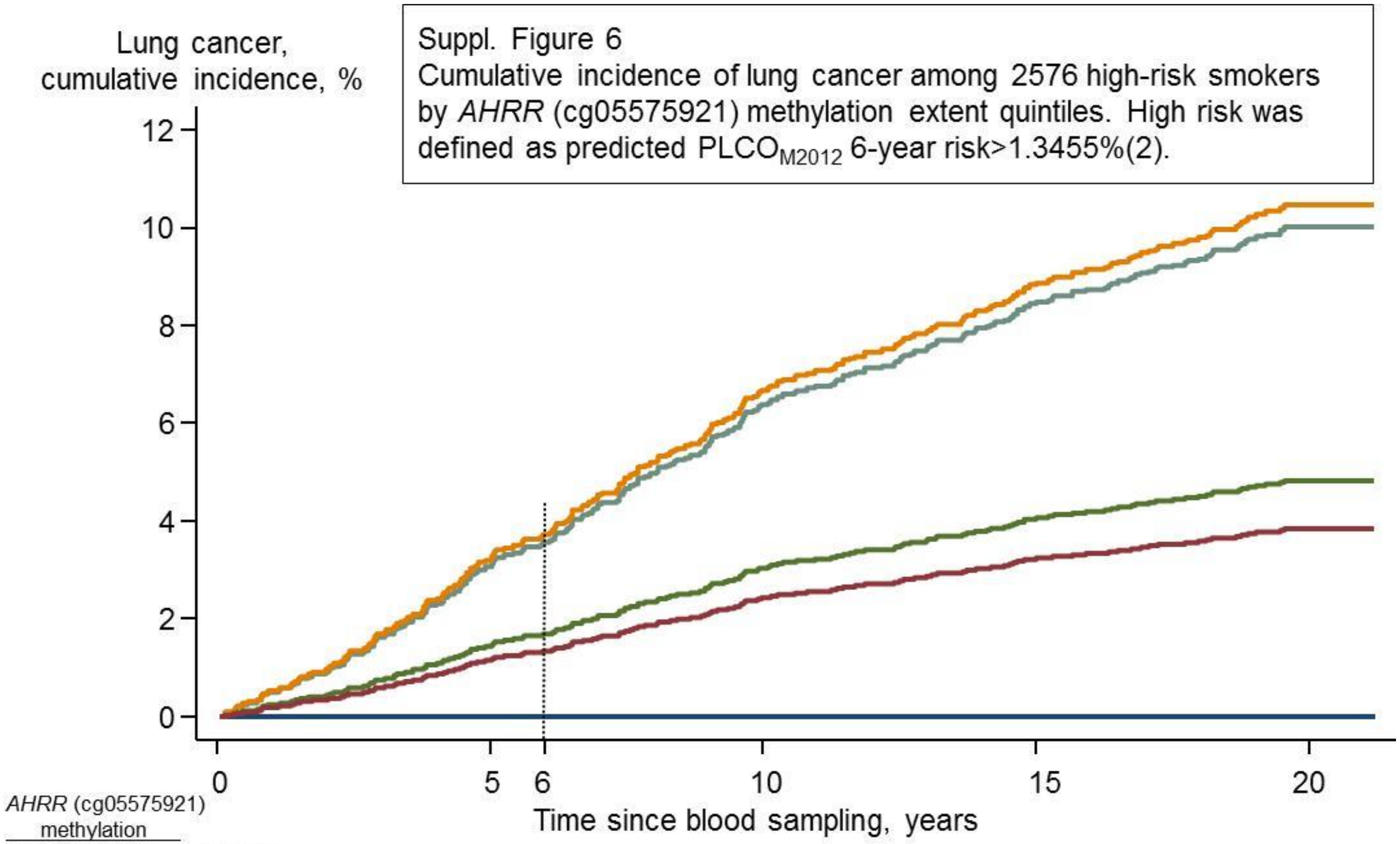

AHRR (cg05575921) methylation Quintile	Extent	Number				
5th	46%	963	768	522	328	70
4th	51%	820	655	452	276	68
3rd	56%	500	390	267	157	26
2nd	62%	198	154	97	63	15
1st	68%	95	71	48	32	8

Suppl. Figure 7
ROC for incident lung cancer by *AHRR* (cg05575921) methylation extent percentage (blue lines) and $PLCO_{M2012}$ predicted risk (black lines). Full lines are among 2576 high-risk smokers, and dashed lines are among 7120 ever smokers. Dotted line is the diagonal.

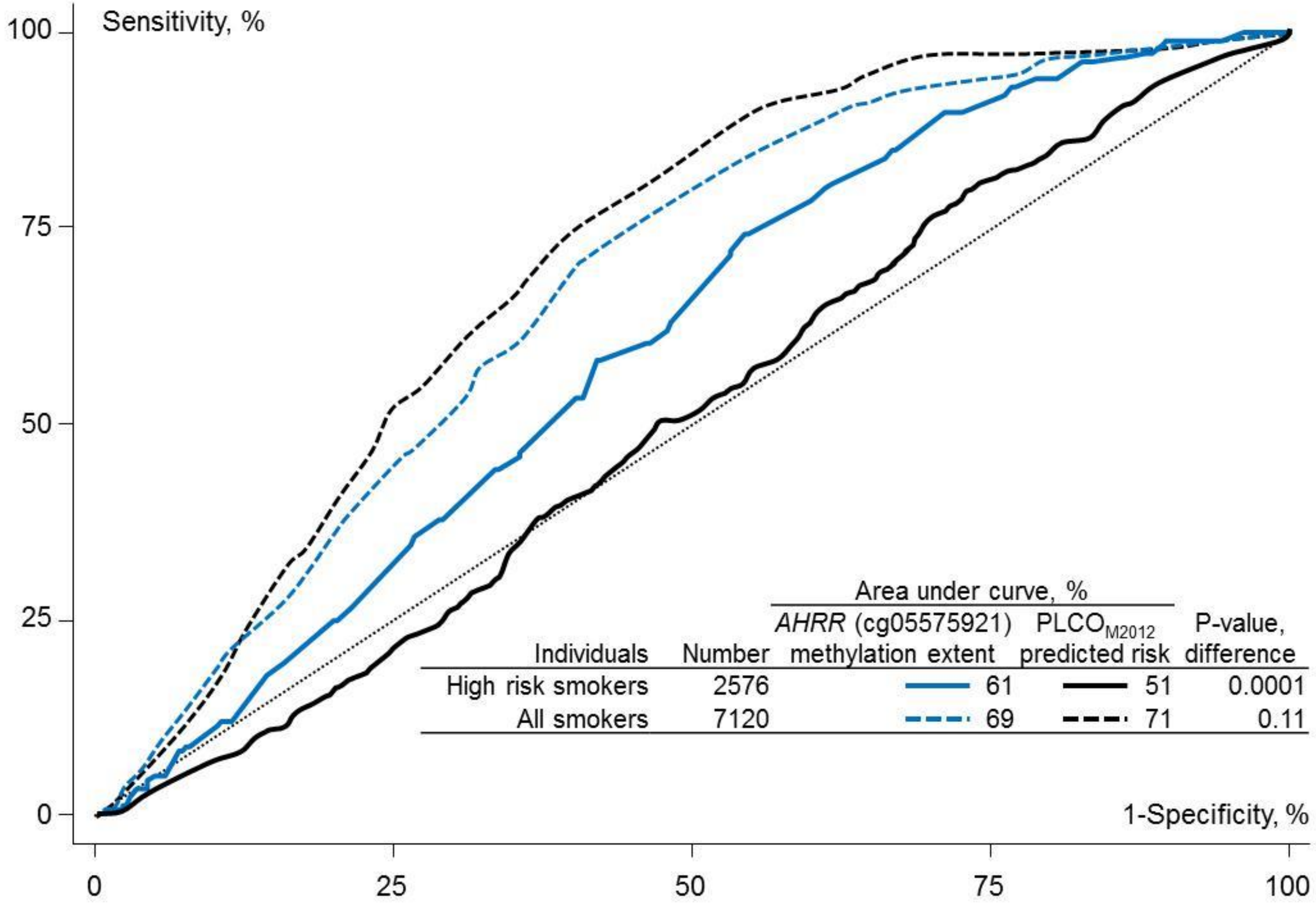

References

1. Dahl M, Tybjaerg-Hansen A, Lange P et al. Change in lung function and morbidity from chronic obstructive pulmonary disease in alpha1-antitrypsin MZ heterozygotes: A longitudinal study of the general population. Ann Intern Med 2002; 136: 270-279.

2. Tammemagi MC, Katki HA, Hocking WG et al. Selection criteria for lung-cancer screening. N Engl J Med 2013; 368: 728-736.
